# Serum concentrations of oxytocin, DHEA and follistatin are associated with osteoporosis or sarcopenia in community-dwelling postmenopausal women

**DOI:** 10.1186/s12877-021-02481-7

**Published:** 2021-10-12

**Authors:** Yanping Du, Cuidi Xu, Hongli Shi, Xin Jiang, Wenjing Tang, Xiaoqing Wu, Minmin Chen, Huilin Li, Xuemei Zhang, Qun Cheng

**Affiliations:** Department of Osteoporosis and Bone Disease, Huadong Hospital affiliated to Fudan University, Research Section of Geriatric Metabolic Bone Disease, Shanghai Geriatric Institute, 221 West Yan An Road, Shanghai, 200040 China

**Keywords:** Biomarkers, Osteoporosis, Sarcopenia, Community-dwelling, Postmenopausal women

## Abstract

**Background:**

Osteoporosis and sarcopenia are major health issues in postmenopausal women due to their high prevalence and association with several adverse outcomes. However, no biomarkers may be used for screening and diagnosis. The current study investigated potential biomarkers for osteoporosis and/or sarcopenia in postmenopausal women.

**Methods:**

A cross-sectional study on 478 healthy community-dwelling postmenopausal women aged 50–90 years was performed. Osteoporosis and sarcopenia were defined according to the World Health Organization (WHO) and Asian Working Group for Sarcopenia (AWGS).

**Results:**

Dehydroepiandrosterone (DHEA) was related to muscle strength (β = 0.19, *p* = 0.041) and function (β = 0.58, *p* = 0.004). Follistatin (β = − 0.27, *p* = 0.01) was related to muscle mass. Oxytocin (β = 0.59, *p* = 0.044) and DHEA (β = 0.51, *p* = 0.017) were related to bone mass. After adjusting for age, oxytocin (odds ratio (OR) 0.75; 95% confidence intervals (CI) 0.63–0.98; *p* = 0.019) was associated with osteoporosis, and DHEA (OR 0.73; 95% CI 0.51–0.96; *p* = 0.032) and follistatin (OR 1.66; 95% CI 1.19–3.57; *p* = 0.022) were associated with sarcopenia.

**Conclusions:**

Postmenopausal women with sarcopenia were more likely to have lower DHEA levels and higher follistatin levels, and postmenopausal women with osteoporosis were more likely to have lower oxytocin levels.

## Background

Osteoporosis and sarcopenia are two common and overlapping geriatric conditions that may lead to a high estimated risk of fractures and a low quality of life in the elderly population [1]. Approximately 212 million people will suffer from osteoporosis, and the total number of hip fractures is forecast to reach 3.25 million in China by 2050 [2]. Because of the severity of the consequences, early screening and diagnosis, prevention and intervention for osteoporosis and the risk of fracture are of great importance.

However, the clinical diagnosis is hampered by three key difficulties in the evaluation of muscle and bone status. First, dual-energy X-ray absorptiometry (DXA), magnetic resonance imaging (MRI) and computed tomography (CT) imaging modalities provide an objective and sufficiently reliable estimation of body composition [3]. However, these imaging techniques are technically complicated and generally only available in well-equipped medical institutions. BIA is a commonly used and feasible tool that is recommended by the AWGS and EWGSOP for community sarcopenia assessment. However, the use of BIA is also limited in elderly individuals, who tend to be dehydrated [4]. Therefore, the muscle mass measured by BIA may be underestimated in elderly individuals because of inadequate hydration. Second, the repeatability of the evaluation methods is poor. The main assessments for muscle function include usual gait speed and the short physical performance battery (SPPB). Technicians generally decide whether the test can be completed and the completion time, which creates a certain bias. Third, osteoporosis and sarcopenia are chronic diseases, and not all individuals exhibit the same rates of muscle and bone loss. Therefore, corresponding indicators to track progression over time or the response to interventions are important and necessary. To overcome the shortage of currently available techniques to evaluate muscle and bone, a pool of serum biomarkers was recently developed based on the molecular biological mechanisms of their involvement in the pathogenesis of sarcopenia and osteoporosis, such as endocrine system, growth factors, and muscle protein turnover. Biomarkers, which theoretically affect these mechanisms, may predict or reflect the state of bones and muscles [5, 6]. However, whether these biomarkers truly reflect the state of bones and muscles was not verified. To explore the relationship between biomarkers and bones and muscles, we identified biomarkers of osteoporosis and sarcopenia according to different pathophysiological mechanisms. (1) Myokines (e.g., myostatin, follistatin, oxytocin, and brain-derived neurotrophic factor (BDNF)). Myostatin is a transforming growth factor-beta (TGF-β) superfamily member and an important negative regulator of skeletal muscle growth [7]. Follistatin is a strong inhibitor of myostatin and acts via activin/myostatin signaling. Myostatin and follistatin are closely related to muscle metabolism and affect bone activities via various pathways [8, 9]. Brain-derived neurotrophic factors and oxytocin are brain-derived neurotrophic factors that were recently found in muscle tissue and mediate muscle regeneration via the cross-talk of skeletal muscle with bone and brain [9]. Oxytocin is mainly produced by the hypothalamus and deposited in the neurohypophysis, supporting maintenance and repair of skeletal muscle, and age-related decline in oxytocin contributes to sarcopenia [10, 11]. However, the relationships of these myokines with bone mass, muscle mass and strength are largely unknown [12]. (2) Sex hormones. Musculoskeletal regulation is generally mediated by mechanical stress stimulation. However, in the absence of load, steroid hormones, such as dehydroepiandrosterone (DHEA), estradiol (E2) and testosterone (T2), increase first, which suggests that the relationship between muscle activity and bone resorption is regulated by sex steroids [13]. Sex hormones are known for their antiaging properties of increasing lean body mass and bone mineral density [14]. Maintaining youthful hormone levels effectively prevents bone and muscle loss and fragility fractures.

We hypothesize that serum myokines and sex hormones are closely related to bone mass, muscle mass and strength and predictive risk factors for bone and muscle loss in the elderly.

The current study enrolled healthy community-dwelling postmenopausal women aged 50–90. We collected history of fragility fracture, evaluated bone mass, muscle mass and strength, and measured sex hormones, myokines and bone turnover markers (1) to examine the changes in these biomarkers with age, (2) examine the relationship between these biomarkers and bone mass, muscle mass and strength, and (3) identify potential biomarkers for the screening and diagnosing of osteoporosis and sarcopenia in postmenopausal women.

## Methods

### Study group

We designed a cross-sectional study by open advertisement from community health services in 2019–2020 to recruit healthy postmenopausal women who had entered menopause for longer than 1 year. After excluding subjects who had a history of amenorrhea (*n* = 8), ovariectomy (*n* = 15), heart disease (*n* = 4), rheumatoid arthritis (*n* = 8), chronic obstructive pulmonary disease (COPD) (*n* = 21), and thyroid disease (*n* = 13), the study cohort included 478 women. All participants were healthy, and none of them suffered from diseases that affected bone metabolism, such as hyperthyroidism, hyperparathyroidism, rheumatoid arthritis, chronic liver or renal disease, malnutrition, or COPD, or took any drugs that affected bone metabolism, e.g., glucocorticoids, heparin, warfarin, thyroxine, sex hormones, bisphosphonates, calcitonin, parathyroid hormone analog, or calcitriol. The Institutional Review Board of Huadong Hospital approved the study protocol (2019 K168). All of the participants signed informed consent before the study began.

We also collected information on fractures that occurred after menopause and 1 year before study entry. Hip fractures and spine fractures were verified by review of medical records and imaging examinations. Details of other fractures were obtained by self-report. According to the National Osteoporosis Foundation [15], fragility fractures are fractures resulting from any fall from a standing height or less.

### Anthropometry

Weight and height were measured when wearing light clothing and without shoes. Body mass index (BMI) was calculated by dividing weight (kg) by the square of height (meter). Assessment criteria of physical exercise and milk consumption were defined based on our previous research in Shanghai [16] and on expert consensus of nutrition and exercise management [17].

1. Physical exercises were defined as running, walking, dancing, tai chi, swimming and ball games. Housework was not considered a form of physical exercise. Physical exercise was assessed in three levels: high level ≥ 30 min/day or an average ≥ 210 min/week; low level < 30 min/day or an average < 210 min/week; and no exercise, which was not performing any of the defined exercises for over 1 year.
2. Milk consumption was assessed based on the following three levels: high level ≥ 250 mL/day; low level < 250 mL/day but ≥50 mL/day; and no consumption < 50 mL/day.
3. Grip strength measurement

A digital handgrip dynamometer (Takei Scientific Instruments, Niigata, Japan) was used to detect grip strength. The participant held the dynamometer up with her elbow joint at a 90-degree angle and squeezed the grip measurement mechanism as hard as she could while watching the screen for a demonstration. The test was typically performed three times with each hand, and the highest value represented the result.

### 6-Meter gait speed

The 6-m gait speed was completed according to AWGS. The subject placed her toes at the starting line of a 6-m course that was clearly demarcated with red tape and walked at normal comfortable walking speed. A handheld timer was started when the subjected lifted one foot, and it was stopped when one of the subjects’ feet struck the ground completely beyond the finish line. This process was performed twice consecutively without rest, and the faster of the 2 times was used for data analysis. Subjects were allowed to use their usual walking aids. Gait speed was calculated by dividing 6 m by the time in seconds required to complete the course.

### BMD and body composition measurements

BMD and body composition were measured using dual-energy X-ray absorptiometry (DXA; Hologic Delphi A; Hologic Inc., Waltham, MA, USA). The same technician performed all scans and analyses. The coefficients of variance (CVs) were 0.86, 1.86 and 0.95% for BMD of the lumbar spine, femur neck and total hip, respectively. The instrument was calibrated daily with a standard phantom.

### Definition of sarcopenia and osteoporosis

Sarcopenia was based on appendicular skeletal muscle mass (ASM; kg) measurements and was normalized for height [ASMI (Appendicular Skeletal Muscle Mass Index) = ASM /height^2^ (kg/m^2^)]. Sarcopenia was defined as meeting the AWGS criteria [18] and categorized using the following threshold values: (1) Low muscle mass was ASMI < 5.4 kg/m2; (2) Low muscle strength was handgrip strength < 18 kg; and (3) Poor physical performance was gait speeds < 0.8 m/s by a 6-m walk test. According to the World Health Organization (WHO) definition [15], osteoporosis was defined as a T-score of BMD ≤ − 2.5 for vertebral, femur neck or total hip, having experienced a low-trauma hip or vertebral fracture, or having osteopenia by BMD who sustained a low-trauma proximal humerus, pelvis, or distal forearm fracture. Osteosarcopenia is the combination of sarcopenia and osteoporosis.

### Laboratory analyses

Serum samples were collected between 0700 and 0900 h after a 10-h fast, and freshly separated serum was divided into 0.5-ml aliquots and stored at − 80 °C. Serum levels of follistatin, myostatin, BDNF and oxytocin were measured using Quantikine ELISA (R&D Systems, Minneapolis, MN, USA), with intra-assay CVs of 2.7–5.9% and interassay CVs of 4.0–7.8%. DHEA was measured using an ELISA kit (Abcam, Cambridge, UK) with intra-assay CVs of 7.9% and interassay CVs of 10.4%. Luteinizing hormone (LH), follicle stimulating hormone (FSH), E2 and T2 were measured using radioimmunoassays (Beckman Coulter, Inc., Brea, CA, USA), with intra-assay CVs of 3.5–4.8% and interassay CVs of 4.4–9.1%. N-terminal propeptide of type 1 collagen (P1NP), C-telopeptide collagen crosslinks (CTX), calciotropic hormone of serum parathyroid hormone (PTH) and 25(OH) D were measured using electrochemical luminescence (Roche Diagnostics, Bostion, MA, USA), with intra- and interassay CVs below 3.5 and 8.4% for CTX and below 2.6 and 4.1% for P1NP, below 2.7 and 6.5% for PTH, and below 7.8 and 10.7% for 25(OH)D.

### Statistics

SPSS v23 (SPSS Inc., Chicago, IL, USA) was used to analyze the data. Kolmogorov Smirnov method was used to test the normal distribution of data. Continuous variables were expressed as the means with standard deviation, median with interquartile range (25–75%), and classification variables were expressed as percentages. Differences between groups were analyzed using ANOVA, Kruskal-Wallis H test or Pearson’s chi-squared test for continuous and categorical variables, respectively. After the residual was tested by Explore, multivariate linear regression models were constructed to analyze the correlation between dependent variables, such as grip strength, gait speed, lean mass, fat mass, ASMI, bone mass, and independent continuous predictors, such as serum myokines, sex hormones, and bone turnover markers. Associations between history of fragility fracture, lifestyle, serum biomarkers and osteoporosis and sarcopenia were analyzed using logistic regression analysis. Osteoporosis and sarcopenia were the dependent variables, and history of fragility fracture and lifestyle and serum biomarkers were the independent variables included in the regression analysis. The results are shown as odds ratios (ORs) with 95% confidence intervals (CIs). Statistical significance was set at *P* < 0.05.

## Results

### Characteristics of study population

The general characteristics of the study subjects are presented in Table 1. A total of 478 postmenopausal women were included in this study. The mean age was 66.77 years, the mean age at menopause was 50.22 years, and the mean BMI was 23.93 kg/m^2^. Ninety-two subjects (19.6%) suffered fragility fractures, and 37 of these subjects had more than one fracture. To analyze the changes in various indicators with aging, subjects were divided into three groups: age65, 65 ≤ age<75, and 75 ≤ age. Grip strength, gait speed, serum levels of DHEA, oxytocin, BMD of femoral neck and total hip, and lean mass decreased significantly with aging. History of fragility fracture and serum level of T2 increased significantly with age. With increasing age, the amount of physical exercise and milk consumption decreased significantly.

**Table 1:** Descriptive characteristics of the studied population

Variable	Age<65 (*n* = 171)	65 ≤ Age<75 (*n* = 185)	75 ≤ Age (*n* = 122)	*P* value
Age (years)	58.0 ± 4.0	69.21 ± 2.9	79.8 ± 3.6	< 0.001
Age of menopause (years)	50.66 ± 4.2	49.89 ± 3.0	50.1 ± 2.9	0.48
BMI (kg/m^2^)	23.94 ± 3.3	23.99 ± 2.9	23.75 ± 4.1	0.95
Grip strength (kg)				
Left	20.18 ± 3.7	18.74 ± 4.1	15.96 ± 4.2	< 0.001
Right	22.88 ± 4.4	21.15 ± 4.0	18.12 ± 4.8	< 0.001
Gait speed (m/s)	1.15 ± 0.19	1.08 ± 0.20	0.87 ± 0.25	< 0.001
Physical exercise (%)				
No	11.8	22.7	48.1	0.008
Low	39.3	38.8	36.1	0.65
High	48.9	38.5	15.8	< 0.001
Milk consumption (%)				
No	33.6	38.3	45.7	0.08
Low	34.3	36.4	38.1	0. 17
High	32.1	25.3	16.2	0.03
History of fragility facture (%)	11.5	20.3	26.6	0.002
LH (lU/l)	20.43 (15.49–24.16)	17.45 (13.10–26.10)	20.67 (16.15–25.25)	0.453
FSH (lU/l)	53.03 (45.73–64.53)	49.16 (41.51–71.78)	56.59 (50.98–80.66)	0.174
E2 (pmol/l)	40.00 (37.00–45.75)	37.00 (37.00–46.00)	39.00 (37.00–62.75)	0.445
T2 (pg/ml)	0.73 (0.59–0.85)	0.67 (0.56–0.88)	0.99 (0.66–1.20)	0.048
DHEA (ng/ml)	47.08 ± 12.3	40.75 ± 11.5	37.0 ± 11.3	0.009
PTH (pg/ml)	38.6 (32.3–47.4)	40.80 (34.38–47.60)	39.9 (27.75–57.70)	0.637
25OHD (ng/ml)	19.75 (14.9–25.85)	21.05 (15.65–27.65)	23.90 (17.80–27.90)	0.562
CTX (pg/ml)	441.65 ± 198.1	365.75 ± 177.6	369.4 ± 187.4	0.07
PINP (ng/ml)	48.18 ± 17.4	43.97 ± 16.4	40.1 ± 14.5	0.12
Follistatin (ng/ml)	14.45 ± 4.9	15.61 ± 4.5	16.9 ± 4.4	0.19
Myostatin (ng/ml)	4.08 ± 1.6	3.61 ± 1.1	3.4 ± 1.5	0.18
Oxytocin (pg/ml)	508.10 (338.33–1245.42)	380.92 (183.96–722.48)	243.53 (191.99–351.53)	0.008
BDNF (ng/ml)	32.06 ± 11.2	29.51 ± 10.4	27.5 ± 11.1	0.33
BMD (g/cm^2^)				
total femur	0.822 ± 0.11	0.765 ± 0.11	0.692 ± 0.14	< 0.001
femoral neck	0.646 ± 0.08	0.612 ± 0.09	0.572 ± 0.12	< 0.001
lumbar spine	0.802 ± 0.12	0.780 ± 0.12	0.751 ± 0.14	0.24
WHR (waist/hip)	1.09 ± 0.16	1.11 ± 0.14	1.09 ± 0.13	0.76
Fat mass (kg)	20.91 ± 4.5	20.73 ± 4.4	19.63 ± 5.2	0.50
Lean mass (kg)	38.06 ± 4.5	36.82 ± 3.6	34.24v4.4	0.001
ASMI (kg/m^2^)	6.25 ± 0.71	6.18 ± 0.56	5.92 ± 0.87	0.13

*LH* Luteinizing hormone, *FSH* Follicle stimulating hormone, *DHEA* Dehydroepiandrosterone, *E2* Estradiol, *T2* Testosterone, *P1NP* N-terminal propeptide of type I collagen, *CTX* Cross-linked C-telopeptide of type I collagen, *PTH* Parathyroid hormone, *25OHD* Serum 25(OH)D, *BDNF* Brain-derived neurotrophic factor, *BMI* Body mass index, *BMD* Bone mineral density, *WHR* Waist/hip ratio, *ASMI* Appendicular skeletal mass index

### Association of serum biomarkers with bone mass, muscle mass or muscle strength adjusted for age

Stepwise multivariate linear regression was performed to assess the correlation between biomarkers and bone mass, muscle mass and strength adjusted for age. The results showed that DHEA was positively related to handgrip (β = 0.403, *p* = 0.041) and gait speed (β = 0.58, *p* = 0.004). Follistatin (β = − 0.28, *p* = 0.01) was negatively related to lean mass, and oxytocin (β = 0.35, *p* = 0.036) was positively related to lean mass. Myostatin (β = 0.92, *p* = 0.021) was positively related to fat mass. Myostatin (β = − 0.31, *p* = 0.032) and follistatin (β = − 0.48, *p* = 0.042) were negatively associated with ASMI. CTX (β = − 0.42, *p* = 0.000) was negatively related to BMD of the spine, and DHEA (β = 0.38, *p* = 0.022) was positively related to BMD of the spine. Oxytocin (β = 0.59, *p* = 0.044) and DHEA (β = 0.52, *p* = 0.017) were positively related to BMD of the total hip, and oxytocin (β = 0.61, *p* = 0.014) was positively related to BMD of the femur neck (Table 2).

**Table 2:** Multivariable linear regression evaluating biomarkers linking to muscle strength, muscle mass, fat mass and bone mass adjusted for age

**Handgrip (right)**	R2	beta	95%CI	*P* value
DHEA	0.611	0.403	0.352 ~ 0.454	0.041
T2	0.005	−1.224	−4.6854-2.236	0.484
E2	0.002	−0.013	− 0.078-0.051	0.682
**Gait speed**		beta	95%CI	*P* value
DHEA	0.234	0.58	0.576 ~ 0.587	0.004
T2	0.006	−1.198	−4.389-1.993	0.458
E2	0.002	0.013	−0.046-0.073	0.657
**Lean mass**		beta	95%CI	*P* value
Follistatin	0.365	−0.28	−0.309 ~ − 0.243	0.010
Oxytocin	0.272	0.35	0.306 ~ 0.398	0.036
**Fat mass**		beta	95%CI	*P* value
Myostatin	0.362	0.92	0.881 ~ 0.960	0.021
**ASMI**		beta	95%CI	*P *value
Myostatin	0.356	−0.31	−0.352 ~ −0.251	0.032
Follistatin	0.227	−0.48	−0.529 ~ − 0.430	0.042
**BMD (lumbar spine)**		beta	95%CI	*P* value
CTX	0.572	−0.42	−0.420 ~ − 0.412	< 0.001
DHEA	0.415	0.38	0.338 ~ 0.414	0.022
T2	0.003	0.025	−0.065-0.114	0.586
E2	0.027	0.001	0.000–0.003	0.123
**BMD (femur neck)**		beta	95%CI	*P* value
Oxytocin	0.347	0.61	0.576 ~ 0.642	0.014
**BMD (total hip)**		beta	95%CI	*P* value
DHEA	0.285	0.52	0.480 ~ 0.551	0.017
T2	0.005	0.031	−0.065-0.127	0.521
E2	0.002	0.001	−0.002-0.001	0.665
Oxytocin	0.366	0.59	0.535–0.651	0.044

*R2* coefficient of determination, *Beta* standardized regression coefficients, *P-value* significant level at *p* < 0.05. *DHEA* Dehydroepiandrosterone, *E2* Estradiol, *T2* Testosterone, *ASMI* Appendicular skeletal mass index, *BMD* Bone mineral density, *CTX* C-telopeptide collagen crosslinks

### Characteristics of demographics, lifestyle and serum biomarkers of participants according to sarcopenia and osteoporosis status

To further demonstrate the association between serum biomarkers and osteoporosis or sarcopenia, all participants were assigned into four groups according to the status of osteoporosis or sarcopenia (Table 3). Of the 478 postmenopausal women, 52 had osteosarcopenia (10.9%), 182 had osteoporosis (38.1%), 51 had sarcopenia (10.6%), and 193 had no sarcopenia and no osteoporosis (NonSP/NonOP) (40.4%). The subjects with osteosarcopenia were older, performed less physical exercise and consumed less milk. These subjects also had lower levels of DHEA, oxytocin and 25OHD, higher levels of follistatin and more history of fragility fracture compared to the subjects in other groups. DHEA (32.51 ± 12.8 and 34.97 ± 16.2 vs. 42.64 ± 12.8 and 48.45 ± 10.6, *p* = 0.042) was significantly lower, and follistatin (18.76 ± 4.8 and 18.97 ± 6.1 vs. 14.93 ± 4.0 and 13.0 ± 4.9, *p* = 0.027) was significantly higher in the sarcopenia group than the respective levels in nonsarcopenia group. Oxytocin was lower in osteoporosis and sarcopenia groups compared to the NonSP/NonOP group, and it was the lowest in osteosarcopenia group. A logistic regression analysis, adjusted for age, demonstrated that history of fragility fracture (no vs. fracture) (OR 0.30; 95% CI 0.05–0.71; *p* = 0.006) and oxytocin (OR 0.75; 95% CI 0.63–0.98; *p* = 0.019) were associated with osteoporosis, and history of fragility fracture (no vs. fracture) (OR 0.45; 95% CI 0.01–0.86; *p* = 0.015), milk consumption (no vs. high) (OR 6.32; 95% CI 1.04–38.29; *p* = 0.045), DHEA (OR 0.73; 95% CI 0.51–0.96; *p* = 0.032), follistatin (OR 1.66; 95% CI 1.19–3.57; *p* = 0.022) and 25OHD (OR 0.51; 95% CI 0.11–0.82; *p* = 0.047) were associated with sarcopenia (Table 4).

**Table 3:** Characteristics of demographic, lifestyle and serum biomarkers of participants according to sarcopenia and osteoporosis status

Group Characteristics	OS (10.9%)	OP (38.1%)	SP (10.6%)	NonSP/NonOP(40.4%)	*P* value
Age (y)	72.8 ± 6.6	65.9 ± 7.3	69.5 ± 10.7	64.5 ± 8.3	0.015
Age of menopause (y)	49.69 ± 3.1	50.51 ± 3.5	49.43 ± 2.3	50.15 ± 3.6	0.76
History of fracture (%)	60.8%	29.8%	32.0%	0	< 0.001
Physical exercise (%)					
No	49.1	41.7	45.6	18.8	< 0.001
Low	31.4	29.3	33.5	32.8	0.023
High	19.5	29.0	20.9	48.4	< 0.001
Milk consumption high (%)					
No	41.6	35.7	38.7	30.3	< 0.001
Low	34.6	39.5	37.9	46.4	< 0.001
High	23.8	24.8	23.4	23.3	0.417
**DHEA (ng/ml)**	32.51 ± 12.8	42.64 ± 12.8	34.97 ± 16.2	48.45 ± 10.6	**0.042**
PTH (pg/ml)	44.5 (29.5–57.2)	40.5 (29.3–55.8)	34.5 (28.3–42.9)	45.4 (32.9–58.5)	0.092
**25OHD(ng/ml)**	21.61 ± 8.121.2 (15.4–28.5)	23.97 ± 9.524.3 (19.1–27.7)	20.54 ± 7.318.3 (13.9–22.7)	25.87 ± 12.226.4 (19.3–35.3)	**0.024**
CTX (pg/ml)	380.83 ± 145.6	425.89 ± 183.19	338.43 ± 159.7	372.60 ± 210.3	0.364
PINP (ng/ml)	36.76 ± 15.2	48.15 ± 17.0	38.62 ± 11.4	44.03 ± 16.5	0.085
**Follistatin (ng/ml)**	18.76 ± 4.8	14.93 ± 4.0	18.97 ± 6.1	13.0 ± 4.9	**0.027**
Myostatin (ng/ml)	3.07 ± 1.3	3.70 ± 1.3	2.75 ± 1.6	3.99 ± 1.5	0.178
BDNF (ng/ml)	22.67 ± 13.3	30.75 ± 10.6	28.74 ± 7.8	30.80 ± 10.9	0.265
**Oxytocin (pg/ml)**	398.3 (173.2–662.6)	425.8 (200.2–702.3)	500.4 (289.3–823.4)	612.7 (356.2–1276.5)	**0.022**
BMD(g/cm2)					
Lumbar Spine	0.703 ± 0.15	0.695 ± 0.06	0.895 ± 0.09	0.886 ± 0.08	< 0.001
Femoral Neck	0.579 ± 0.09	0.612 ± 0.10	0.648 ± 0.06	0.659 ± 0.10	0.019
Total Hip	0.722 ± 0.10	0.748 ± 0.11	0.767 ± 0.09	0.816 ± 0.13	0.007
BMI (kg/m2)	19.41 ± 1.9	23.96 ± 3.3	20.31 ± 1.9	25.09 ± 2.6	< 0.001
Fat mass (kg)	15.50 ± 2.3	20.86 ± 4.9	18.59 ± 5.1	21.43 ± 4.0	0.001
Lean mass (kg)	30.43 ± 2.4	36.99 ± 3.8	32.87 ± 3.1	38.27 ± 3.8	< 0.001
ASMI (kg/m2)	4.92 ± 0.2	6.22 ± 0.5	4.96 ± 0.3	6.44 ± 0.5	< 0.001

*OS* Osteosarcopenia, *OP* Osteoporosis, *SP* Sarcopenia, *NonSP/NonOP* No sarcopenia no osteoporosis, *DHEA* Dehydroepiandrosterone, *PTH* Parathyroid hormone, *CTX* C-telopeptide collagen crosslinks, *P1NP* N-terminal propeptide of type 1 collagen, *BDNF* Brain-derived neurotrophic factor, *BMD* Bone mineral density, *BMI* Body mass index, *ASMI* Appendicular skeletal mass index

**Table 4:** Correlation of history of fragile fracture, lifestyle and serum biomarkers with osteoporosis or sarcopenia by Logistic Regression

Variables	Osteoporosis		Sarcopenia	
	OR(95% CI)	*P* value	OR(95% CI)	*P*-value
**History of fragile fracture** (no vs. fracture)	0.30 (0.05–0.71)	**0.006**	0.45 (0.01–0.86)	**0.015**
Physical exercise (no vs. high)	0.97 (0.44–3.13)	0.94	1.07 (0.24–2.51)	0.69
**Milk consumption** (no vs. high)	1.39 (0.57–3.34)	0.46	6.32 (1.04–38.29)	**0.045**
**DHEA (ng/ml)**	0.75 (0.61–1.05)	0.06	0.73 (0.51–0.96)	**0.032**
T2	0.89 (0.63–1.24)	0.62	0.78 (0.44–2.22)	0.72
E2	0.67 (0.33–2.65)	0.58	0.90 (0.46–3.12)	0.65
PTH (pg/ml)	1.01 (0.97–1.04)	0.64	0.97 (0.90–1.04)	0.36
**25OHD(ng/ml)**	0.98 (0.93–1.07)	0.22	0.51 (0.11–0.82)	**0.047**
CTX (pg/ml)	1.02 (0.98–1.05)	0.17	1.02 (0.92–1.12)	0.78
PINP (ng/ml)	1.12 (0.97–1.48)	0.51	1.07 (0.95–1.17)	0.31
**Follistatin (ng/ml)**	1.08 (0.56–2.09)	0.17	1.66 (1.19–3.57)	**0.022**
Myostatin (ng/ml)	0.95 (0.90–1.13)	0.82	1.01 (0.98–1.03)	0.56
BDNF (ng/ml)	0.78 (0.38–1.62)	0.50	0.35 (0.07–1.82)	0.35
**Oxytocin (pg/ml)**	0.75 (0.63–0.98)	**0.019**	0.88 (0.57–1.01)	0.14

Multivariate logistic analysis was performed after adjusting for age. *DHEA* Dehydroepiandrosterone, *PTH* Parathyroid hormone, *CTX* C-telopeptide collagen crosslinks, *P1NP* N-terminal propeptide of type 1 collagen, *BDNF* Brain-derived neurotrophic factor

## Discussions

Using cohorts of community-dwelling postmenopausal women in Shanghai, China, we examined the relationship between 13 circulating biomarkers, including DHEA, E2, T2, LH, FSH, myostatin, follistatin, oxytocin, BDNF, CTX, PINP, PTH and 25OHD, and bone mass, muscle mass, strength and function to evaluate the practical value of these biomarkers in clinical practice.

Follistatin positively correlated with LH and FSH and negatively correlated with bone mass, muscle mass and strength. Our results showed that increased follistatin coexisted with reduced muscle strength and low BMD in patients, which is consistent with Fife [8], who reported that circulating myostatin and follistatin were negatively associated with muscle mass and function in elderly women. Myostatin is a main negative regulator of skeletal muscle growth, and follistatin is a strong inhibitor of myostatin-mediated muscle wasting that increases muscle mass and muscle regeneration and modulates bone metabolism by affecting activin/myostatin signaling. However, follistatin levels negatively correlated with muscle mass in our study. The accelerator-brake hypothesis may explain this phenomenon [19]. High follistatin expression has been observed in response to unfavorable metabolic environments, which may be induced by low muscle mass and function. However, high follistatin inhibits myostatin-mediated muscle wasting [20]. We also found that myostatin positively correlated with fat mass. Several lines of evidence suggest that obesity poses a threat to skeletal muscle health via myostatin [21]. Extremely obese women secrete and express increased amounts of myostatin in skeletal muscle, which correlate with insulin resistance [22]. The expression of myostatin decreased significantly decreased after gastric surgery, and insulin resistance was significantly improved [23]. This evidence supports the hypothesis that obesity leads to an increase in myostatin, which impairs skeletal muscle health.

The main strength of the current study was that it simultaneously considered the relationship between several biomarkers, including myostatin, follistatin, oxytocin, BDNF, DHEA, T2 and E2, and osteoporosis and/or sarcopenia in the same study population. Therefore, the degree of correlations between different biomarkers and bone and muscle were compared.

For the biomarkers related to sex hormones, our study showed that DHEA was positively related to handgrip and gait speed. Further results showed that postmenopausal women with sarcopenia were more likely to have higher DHEA levels. However, T2 and E2 were not related to muscle mass, grip strength or gait speed. These results suggested that the circulating sex hormones DHEA was a better biomarker than T2 and E2 for muscle strength and gait speed, which likely occurred because DHEA is a precursor for sex steroids. Three reasons may support this hypothesis. First, DHEA does not bind to sex hormone-binding globulin (SHBG), which suggests that DHEA has free access to target organs compared to SHBG-bound E2and T2 [24]. Second, DHEA is an androgen prehormone produced in the zona reticularis of the female adrenal gland and ovarian theca cells, and it acts as an upstream precursor of T2 and E2 in postmenopausal women [25]. DHEA in postmenopausal women becomes the predominant sex hormone instead of E2 [26]. Circulating DHEA provides substrates that are required for conversion into potent androgens and estrogens in peripheral tissues. Skeletal muscles are capable of synthesizing androgens and estrogens from DHEA [27]. Therefore, T and E2 levels in serum do not represent the concentrations of T and E2 in local muscle tissue and tends to underestimate their effects on muscle.

Our study also showed that oxytocin was independently associated with osteoporosis, excluding the effects of estrogen, androgen and DHEA, which suggested that the effect of oxytocin on bone did not completely depend on the levels of sex hormones. Osteoblasts produce oxytocin under the control of estrogen via a nongenomic mechanism [28]. Oxytocin mediates the anabolic action of estrogen on the skeleton [29]. However, several studies showed that oxytocin had a direct effect on osteoblasts and osteoclasts without estrogen in vitro and ex vivo [30, 31]. Lawson reported that a decrease in nocturnal oxytocin secretion in amenorrheic athletes was strongly associated with a change in bone architecture after controlling for estradiol [32], which suggests that oxytocin plays a role in the rescue of bone metabolism in postmenopausal women with low estrogen levels.

Osteoporosis, sarcopenia and osteosarcopenia are common skeletal and muscle diseases in the elderly. To further verify the clinical application value of biomarkers, we compared serum biomarker levels in the four groups of postmenopausal women with different bone muscle statuses and analyzed the relationships of biomarkers with the risk of osteoporosis and sarcopenia. The results showed that elevated oxytocin levels were associated with a reduced risk of osteoporosis, and elevated DHEA levels were associated with a reduced risk of sarcopenia. However, elevated follistatin levels were associated with an increased risk of sarcopenia. The current study found that DHEA and oxytocin were significantly lower in postmenopausal women with a history of fragility fracture compared to women without fracture (data not shown). Therefore, serum DHEA and follistatin may be biomarkers of sarcopenia, and serum oxytocin may be a biomarker of osteoporosis.

Several studies showed that physical exercise influenced serum levels of follistatin and DHEA [33–35]. Our results showed that postmenopausal women without osteoporosis/sarcopenia performed higher exercise levels than women with osteoporosis/sarcopenia. For these results, we considered that the positive promoting effect of exercise on muscle and bone may occur via changes in the expression of these factors, including follistatin and DHEA, during exercise. Therefore, the changes in these factors may be an intermediate link in the impact of exercise on muscle and bone. However, our results also showed that follistatin and DHEA were associated with sarcopenia, excluding the influence of exercise.

Our results showed that vitamin D deficiency was very common in the Chinese population. We previously published a relevant article [16]. Many studies showed that vitamin D was closely related to muscles and bones. Notably, our results showed that excluding the influence of vitamin D, oxytocin was also associated with osteoporosis, and follistatin and DHEA were associated with sarcopenia.

In our study, milk consumption (< 50 mL/day vs. ≥ 250 mL/day) (OR 6.32; 95% CI 1.04–38.29; *p* = 0.045) was associated with sarcopenia. The reason is that milk contains nutrients, especially whey protein, that may be myoprotective. One trial investigated the effect of adding milk protein to the habitual diet on skeletal muscle mass, strength, and physical performance in Mexican elderly individuals without sarcopenia. The results showed that consumption may reduce the risk of sarcopenia by improving skeletal muscle mass due to the addition of nutrient-rich dairy proteins to the habitual diet [36]. However, current evidence does not show beneficial effects of milk on muscle health in older adults. This discrepancy may be due to high habitual protein intakes (> 1.0 g/kg BW/d) in study participants [37]. Our study did not calculate the total habitual protein intake of the subjects, and the results have certain limitations.

Our study has several other limitations. First, we recruited healthy postmenopausal women from community health services. Therefore, the conclusions from our data may not be applicable to men and unhealthy individuals. Second, this cross-sectional study does not allow us to obtain causal relationships. Longitudinal studies should be performed to further examine the predictive effect of circulating biomarkers for osteoporosis and sarcopenia.

In summary, the current study is the first study to explore the relationships between serum myokines, sex hormones, bone turnover markers, bone mass, muscle mass, and muscle strength simultaneously in the same study population. We observed that postmenopausal women with sarcopenia were more likely to have lower DHEA levels and higher follistatin levels, and postmenopausal women with osteoporosis were more likely to have lower oxytocin levels. Notably, the correlations between serum follistatin and DHEA and sarcopenia and the correlation between serum oxytocin and osteoporosis were independent of exercise and vitamin D levels. Therefore, serum oxytocin, DHEA and follistatin are promising candidates as serum biomarkers related to osteoporosis and/or sarcopenia, regardless of exercise and vitamin D status.

## Data Availability

The datasets used and/or analysed during the current study are available from the corresponding author on reasonable request.

## Abbreviations

ASM: Appendicular skeletal mass
ASMI: Appendicular skeletal mass index
AWGS: Asian Working Group for Sarcopenia
BDNF: Brain-derived neurotrophic factor
BMD: Bone mineral density
BMI: Body mass index
CI: Confidence intervals
COPD: Chronic obstructive pulmonary disease
CT: Computed tomography
CTX: C-telopeptide collagen crosslinks
CV: Coefficients of variance
DHEA: Dehydroepiandrosterone
DXA: Dual energy X-ray absorptiometry
E2: Estradiol
FSH: Follicle stimulating hormone
LH: Luteinizing hormone
MRI: Magnetic resonance imaging
OR: Odds ratios
P1NP: N-terminal propeptide of type 1 collagen
PTH: Parathyroid hormone
SHBG: Sex hormone-binding globulin
SPPB: Short physical performance battery
T2: Testosterone
TGF-β: Transforming growth factor-beta
WHO: World Health Organization
